# The Impact of Fruit Etiolation on Quality of Seeds in Tobacco

**DOI:** 10.3389/fpls.2020.563971

**Published:** 2020-10-08

**Authors:** Domenica Farci, Patrycja Haniewicz, Emma Cocco, Antonio De Agostini, Pierluigi Cortis, Magdalena Kusaka, Maria C. Loi, Dario Piano

**Affiliations:** ^1^Department of Plant Physiology, Warsaw University of Life Sciences—SGGW, Warsaw, Poland; ^2^Laboratory of Plant Physiology and Photobiology, Department of Life and Environmental Sciences, University of Cagliari, Cagliari, Italy; ^3^Department of Life and Environmental Sciences, University of Cagliari, Cagliari, Italy

**Keywords:** dormancy, germination, *Nicotiana tabacum*, phytohormones, photosynthesis, post-anthesis, ripening, photomorphogenesis

## Abstract

Seed’s maturity and integrity are essential requirements for germination, and they rely on nutrients availability and a correct phytohormones’ balance. These aspects are prerequisites for prompt germination at the end of the dormancy period and strictly depend on chloroplast metabolism and photosynthesis. In the present work, capsules of *Nicotiana tabacum* were grown in dark during the whole post-anthesis period. Among others, photosynthetic rates, dormancy, and phytohormones levels in seeds were found to be significantly different with respect to controls. In particular, etiolated capsules had expectedly reduced photosynthetic rates and, when compared to controls, their seeds had an increased mass and volume, an alteration in hormones level, and a consequently reduced dormancy. The present findings show how, during fruit development, the presence of light and the related fruit’s photosynthetic activity play an indirect but essential role for reaching seeds maturity and dormancy. Results highlight how unripe fruits are versatile organs that, depending on the environmental conditions, may facultatively behave as sink or source/sink with associated variation in seed’s reserves and phytohormone levels.

## Introduction

Fruits and seeds are the key players in plant reproduction and species dynamics (Hamilton and May, 1977; Springthorpe and Penfield, 2015). Their properties and functions are intimately related and follow the same fate (Hamilton and May, 1977; Aharoni and O’Connell, 2002; Springthorpe and Penfield, 2015). Immediately after anthesis, flower pollination and fruit formation take place, resulting in seeds (pre-) maturation while the fruit ripens (Sumner and Mollon, 2000).

Fruit ripening and seed development are processes strictly depending on the photosynthates trafficking (Kourmpetli and Drea, 2014). In this respect, fruits are unique organs that are both centers of photosynthates accumulation, behaving as sinks and sources of photosynthates due to their photosynthetic parenchyma (Lytovchenko et al., 2011). This bivalent behavior starts from the early stages of fruit formation, lasts immediately until ripening (Yamaki, 2010), and appears to be important for seeds development (Lytovchenko et al., 2011). Accordingly, the photosynthates accumulation is shared between leaves and developing-unripe fruits, resulting in a characteristic species-specific profile of their relative contribution (Hidaka et al., 2019). In fruits, the ratio between their own contribution and the one from leaves strictly depends on their photosynthetic capabilities. Considering that fruits are also sink organs with the ability to increase the concentration of photosynthates and solutes by their own, leaves are a subordinated source for photosynthates production according to the Münch scheme (Christy and Ferrier, 1973). On one hand, by allocation mechanisms, the photosynthates from leaves are promptly translocated to the fruits to an extent that is defined by the fruit itself, the species, and the environmental conditions (Hidaka et al., 2019). On the other hand, if the photosynthates production from fruits and the related coordination role are obvious, it is not obvious their precise contribution with respect to leaves (Baïram et al., 2019). Accordingly, for a given species, the leaves contribution is primarily expected to depend on the fruits’ photosynthetic capabilities, hence on the extent and efficiency of the fruit photosynthetic parenchyma, as well as the final mass to be reached. Related to this, it must be also noticed that while fruit growth takes place, the surface of fruits increases proportionally but the number of stomata remains the same (Hiratsuka et al., 2015), suggesting that the leaves contribution increases while the ripening is approached. In fact, the resulting reduction of stomata’s density brings to a decreased efficiency in gas exchanges and related photosynthetic rates (Hiratsuka et al., 2015). This causes an increased difference in solutes concentration between sink and sources; hence, it brings to an increased flow of photosynthates from leaves to fruits (Christy and Ferrier, 1973). Under this complex phenomenology, it is evident that a fine reciprocal regulation is needed to harmonize the photosynthetic activities of these two districts and their related productions (Baïram et al., 2019). This role of coordination is pivotally modulated by phytohormones that tune an efficient photosynthate trafficking and allocation (Robert, 2019). In this study, immediately after pollination, flowers were shielded from light to induce etiolation. The etiolated capsules presented expectedly reduced photosynthetic rates and significative differences when compared to controls, especially with respect to seeds properties. Under etiolated conditions, the contribution of photosynthates from external sources is dominant, and the differences in seeds and fruits allow to infer about the contribution of each source in normal light conditions, eventually providing evidence for the essential role of balancing and regulation played by the fruit during development. Here, the etiolation of tobacco capsules, which were subjected to targeted light starvation, provides evidence about the primary role played by the photosynthetic activity in green-unripe fruits. This role appears strongly mediated by phytohormones that act by driving fruit development and demonstrates how photomorphogenesis is essential in fruits for reaching the seed’s maturity and dormancy.

## Materials and Methods

### Growth and Cultivation of Tobacco Plants

Seeds of *Nicotiana tabacum* (cv. *Petit havana*) were imbibed in tap water by incubating them for 24 h at 4°C under dark conditions. After imbibition, seeds were sowed, and plants were grown for 15–20 weeks under a constant temperature of 25°C, 50% relative humidity, a light regime of 12 h/day, and a light intensity of 150–200 μmol photons/(s·m^2^).

### Induction of Etiolation

At their early developmental stage, the etiolation of capsules was induced as follows. At the 2nd day post-anthesis (DPA), when pollination has already taken place and the ovary started to swell, the senescent corolla was removed, and the green ovary was covered till the insertion of the peduncle on the inflorescence axis by a triple-layered pocket made of black-crepe paper. This paper shielded the ovary from light but allowed the gas exchange between the ovary and the environment. The protective coverage was maintained in place for 2–3 weeks, allowing the period of swelling and seeds’ maturation to take place under dark conditions.

Similar experiments were extended to the whole leafage; the whole plant was covered with a single pocket of black-crepe paper, leaving only the inflorescence uncovered, but flowers were abscised after 2–5 DPA.

Control plants, which were plants not subjected to etiolation in any of their parts, provided capsules and seeds for controls data (“external controls”). Uncovered capsules from test plants (plants subjected to etiolation in some of their parts) were also used as “internal controls.” In this case, neither capsules nor seeds did show significant differences with respect to “external controls” (see following paragraphs).

Experiments were performed on a pool of three test plants and three control plants.

### Photosynthetic Measurements

The photosynthetic activity on etiolated and control capsules was recorded by using a Mini PPM 100 fluorimeter (EARS, The Netherlands). This handy fluorimeter is provided with a holed spacer (~0.5 cm diameter) that allows measurements on a very small photosynthetic surface. In these conditions, the measurement on a flat surface (e.g., leaf) or a slightly bent surface (e.g., capsules) does not affect the efficiency of the measurement and makes the instrument ideal for this specific need. After 15 min of dark adaptation, a pulse width modulation (PWM) of 7.2 kHz at 455 nm was provided. Each saturating pulse carried about 2,500–3,000 μmol photosynthetically active radiation (PAR) in an ambient, where the resulting intensity was of about 5–15 μmol PAR. The fluorescence was recorded with an infrared optical bandpass filter in the range of 700–780 nm. The maximum fluorescence (F_m_), the fluorescence in ambient light (F_0_), and the photosynthetic yield (Y) were used as a discriminant of the photosynthetic activity (De Agostini et al., 2020) between etiolated capsules and controls, with the latter represented by leaves and non-etiolated capsules. All the measurements were performed in three repetitions, each in a different leaf or capsule from three different plants, finally providing a dataset of nine measurements shown here as mean ± SD for each studied condition. The means between control and test samples were compared and weighted according to the *t*-test. No differences were observed between “internal” and “external” control experiments (see section Induction of Etiolation). However, the data shown for these experiments are referred to the internal controls.

### Germination Tests

Germination tests were performed on 30 and 90 DPA seeds in three repetitions of 50 seeds each for etiolated and control capsules from three different individuals. After imbibition, seeds were sowed by equally spreading them on a 20-cm diameter pot filled with opportunely-wetted soil as described in section Growth and Cultivation of Tobacco Plants. After sowing, germination was monitored daily for the following 30 days, and it was recorded and expressed by three basic indexes: the total number of germinated seeds (*n*_t_) expressed in % (*N*_t_); the partial number of germinated seeds (*n*_p_) from the last check, also expressed in % (*N*_p_); and the % of mortality (*M*) estimated at the end of the experiment. The indexes’ percentages were referred to the total number of seeds sowed. The three parameters were defined as: *N*_t_ = (*n*_t_/50) × 100; *N*_p_ = [(*n*_t_ − *n*_t − 1_)/50] × 100; and *M* = 1 − (*N*_t_), where *n*_t_ is the number of germinated seeds at a given time, *n*_t − 1_ is the number of germinated seeds at the last but one measurement, and 50 is the number of sowed seeds. To better appreciate the trend, the collected data were adapted to a logistic curve using the software mycurvefit,^1^ and the obtained equation was graphically represented using the software PLOT 2.^2^ In the plot, each daily value is given as mean ± SD of three independent germination experiments performed in the same conditions for seeds collected from etiolated and control capsules. We considered as germinated each seed having both the primary root and the cotyledons protruded. These two main criteria were assessed as follows: after sowing, the white primary root emitted by germinated seeds could be easily identified in the dark background provided by the soil; and a few days after, the cotyledon leaves could be also observed, confirming the “root-count.” No differences were observed between “internal” and “external” control experiments (see section Induction of Etiolation), thus the data shown here are referred to the latter unless otherwise specified.

### Seeds and Capsules Biometrics

Experiments were performed on a pool of three plants, with three independent measurements for each plant providing a dataset of nine values for each parameter: capsule weight, total seeds weight per capsules, averaged seed weight, empty capsule weight (capsule without seeds), seeds length, and seeds width. This design was followed for the etiolated and control capsules. No differences were observed between “internal” and “external” control experiments (see section Induction of Etiolation), and thus, the data shown here are referred to the latter unless otherwise specified. Concerning the weight and dimensions of the seeds, measurements were performed on a sample of 50 seeds under the schemes described in the previous paragraphs (three samples from the same plant and the whole repeated for three different plants). The weight of seeds and capsules was measured using an analytical balance (readability ± 0.01%) at 20°C and 70% relative humidity. The averaged values obtained for each parameter are given as mean ± SD. For a given parameter, the means between control and test samples were compared and weighted according to the *t*-test. The dimensions of seeds were measured using imageJ (Schneider et al., 2012).

### Analysis of Seed’s Phytohormones and Their Metabolites by Mass Spectrometry

For quantitative measurement of phytohormones, 45 days old seeds from etiolated and control capsules were collected. Prior analyses, the seeds were kept in dark at 20°C. The analysis was performed as previously reported (Mahalingam, 2019), with some minor modifications. Briefly, for each sample, 200 mg of seeds were quickly washed with double distilled water and dried down in a 2 ml Eppendorf tube. Phytohormones were extracted using cold methanol, while deuterium-labeled internal standards (a mixture of D5-IAA, D6-ABA, D4-SA, D2-JA at 1.25 μM each, and D2-GA1 at 2.5 μM – Sigma Aldrich, St. Louis) were solubilized in acetonitrile (50, 50, v/v) and used as marker. After shaking for 25 min, the sample was centrifuged at 16,000 *g* for 10 min. The obtained supernatant was stored into a new tube, and the pellet was subjected to a second extraction. These two supernatants were pooled and dried down using a speed-vac. Pellets were re-dissolved in 200 μl of 15% methanol. The liquid chromatography (LC) separation was performed with a ZORBAX Eclipse Plus C18 column (2.1 × 100 mm, Agilent) at 0.45 ml/min flow rate. The mobile phases A (0.1% formic acid) and B (0.1% formic acid/90% acetonitrile) were mixed according to the following gradient: 5% B for 1 min, to 60% B in 4 min, to 100% B in 2 min, hold at 100% B for 3 min, to 5% B in 0.5 min. Phytohormones levels were quantified using a Shimadzu LC system interfaced with a Sciex QTRAP 6500+ mass spectrometer equipped with a TurboIonSpray (TIS) electrospray ion source. The sample acquisition and data analysis were controlled by the Analyst software (version 1.6.3). Tuning and calibration of the QTRAP 6500+ mass spectrometer were performed according to the manufacturer’s recommendations. Phytohormones were detected using multiple reaction monitoring (MRM) transitions that were optimized using the standards. The instrument was set-up to acquire in positive and negative ion switching. For phytohormones’ quantification, an external standard curve was prepared using a series of standards with different concentrations of unlabeled phytohormones and fixed concentrations of the deuterium-labeled standards. Eventually, the data normalization was based on the internal standards: D5-IAA, D2-JA, D4-SA, D6-ABA, and D2-GA_1_ to account for both experimental variation and hormone extraction/ionization efficiency. The amounts of the detected phytohormones were expressed in ng/g. Measurements were performed in three repetitions from different plants. The averaged values are given as mean ± SD. The means between control and test samples were compared and weighted according to *t*-test.

### Statistics

Whole measurements were performed in nine independent repetitions (three samples from the same plant and the whole repeated for three different plants) for each condition (etiolated and control). Mass spectrometry (MS) analysis and germination tests were performed in three repetitions each one on seeds from a different plant. All the values resulting from each dataset are given as mean ± SD. Statistical tests were performed using GraphPad^3^ with *t*-test analysis. Statistical significance of differences was estimated by the *p* relative to each test and control pool for a given parameter.

## Results

### Etiolated Capsules Complete Their Maturation and Their Fate Depends on Leaves

**Figure 1:**
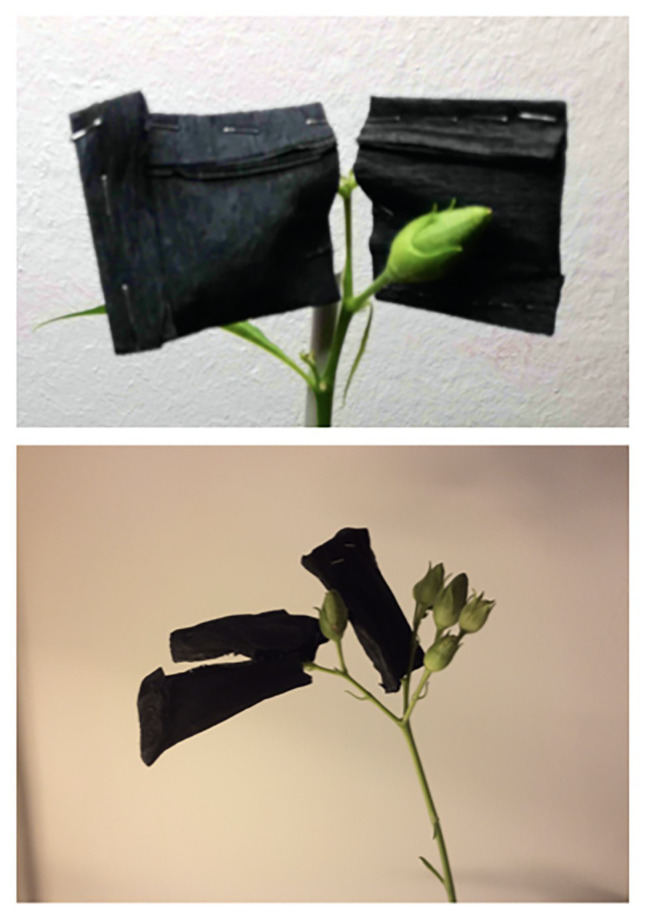
Tobacco capsules were covered to induce etiolation. At the 2nd DPA, a pocket consisting of a triple layer of black-crepe paper was applied. This setup allowed the development and maturation of the fruits in the absence of light.

**Figure 2:**
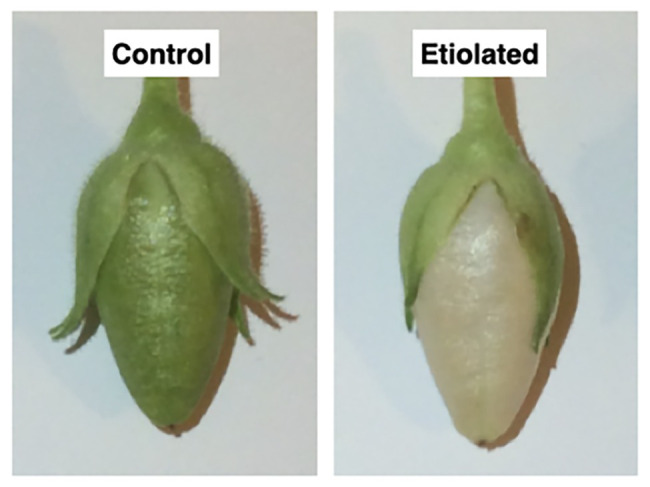
Morphological differences between dark-developed and light-developed capsules (controls). Both capsules grew normally with the only difference ascribable to the color; the control capsules (grown under normal light) were green, while the test capsules (grown in the dark) underwent etiolation.

To assess their own photosynthetic contribution, tobacco capsules were covered at the 2nd DPA by applying a pocket consisting of a triple layer of black-crepe paper (Figure 1). This setup prevented the passage of light and allowed to test fruit maturation under dark conditions. In these conditions, fruit development and maturity took place in 15–20 DPA without any major difference compared to controls. The only exception was that dark-grown capsules underwent etiolation (Figure 2).

### Etiolated Capsules Carry Photosynthesis With Low Rates

Dark-developed capsules appeared etiolated (Figure 2) in their carpel region. On the contrary, sepals maintained the typical photosynthetic parenchyma even if they were also kept in the dark, suggesting different regulation mechanisms with respect to photomorphogenesis/skotomorphogenesis (Figure 2). Given the light-green color of the etiolated capsules, we investigated whether they may perform photosynthesis and to which extent. Basic photosynthetic parameters measured on etiolated capsules were compared with control capsules and leaves from the same plants (Table 1). In particular, the estimated maximum efficiency of PSII [*Y* = (F_m_ − F_0_)/F_m_ = 1 − (F_0_/F_m_)] was used as a reference for the photosynthetic rate, assuming an ideal situation, where the whole PSII reaction centers in the sample are fully open. These values were found to be low in dark-developed capsules (*Y* = 48.40 ± 0.34%), while control capsules showed rates (*Y* = 67 ± 0.53%) roughly comparable to leaves (*Y* = 71.60 ± 0.61%). Lower *Y* values of control capsules with respect to leaves values must be interpreted considering the morpho-structural adaptation of leaves to maximize light capture and photosynthetic efficiency. Consistently, the F_0_/F_m_ ratio, which is an indication of fluorescence not related to photochemistry, was observed to be comparable for leaves and control capsules, with values of 0.28 ± 0.01 and 0.33 ± 0.01, respectively, while the dark-developed capsules presented almost two times higher values (~0.52 ± 0.01). These values for the etiolated capsules suggested a low efficient photosynthetic apparatus and confirmed a dominant etiolated profile (Table 1; *p* < 0.0001). Analysis of leaves and not-etiolated capsules from control plants (external controls, see section Induction of Etiolation) and test plants (internal controls, see section Induction of Etiolation) were comparable (data not shown).

**Table 1:** Measures of the main photosynthetic parameters on dark-grown capsules and controls (leaves and light-developed capsules).

	Etiolated capsules (dark-developed)	Control capsules (light-developed)	Leaves (internal control)
F_0_^*^	150.33 ± 2.78	357.11 ± 7.15	462.89 ± 11.24
F_m_^*^	291.22 ± 5.91	1084.67 ± 8.82	1637.78 ± 26.34
F_0_/F_m_^*^	0.52 ± 0.01	0.33 ± 0.01	0.28 ± 0.01
*Y* (%)^*^	48.4 ± 0.34	67 ± 0.53	71.6 ± 0.61

The fluorescence in ambient light (F_0_) and the maximum fluorescence (F_m_) are the fluorescence level of dark-adapted sample when all the photosystem II reaction centers are open or closed, respectively; *Y* is the photosynthetic yield; and F_0_/F_m_ an indication of fluorescence not related to photochemistry. For each condition, data were expressed as mean ± SD on nine independent measurements from three different individuals. The *p* indicate statistical significance for a given measurement between etiolated and control capsules.

* Indicates a value of *p* < 0.0001 between etiolated and control capsules for a given parameter.

### Seeds of Etiolated Capsules Are Heavier and Bigger Respect to Controls

**Figure 3:**
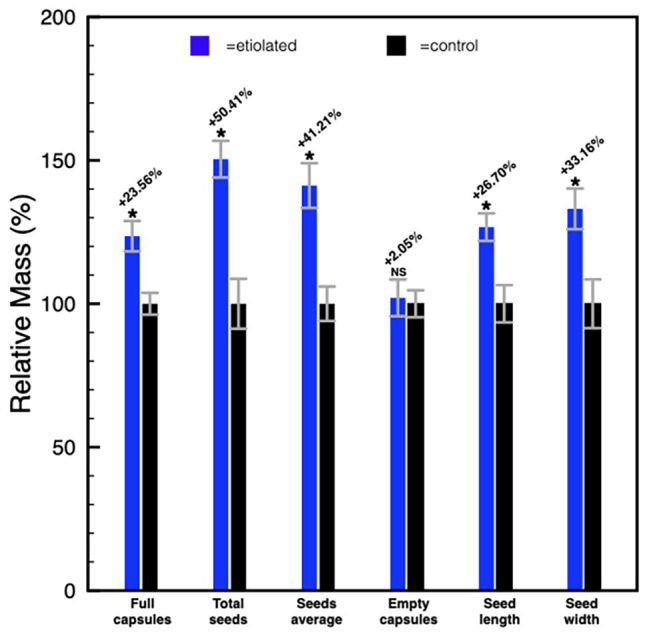
Histogram representing the differences between etiolated and control capsules. Full capsules, seeds, and empty capsules were measured and the differences were expressed as a relative mass with respect to controls. Values are expressed as mean ± SD on nine capsules and on their related total seeds content from three different plants. Mean ± SD of seeds’ mass and dimensions were calculated from nine replicas of 50 seeds collected from nine different capsules of three different plants. *p* indicate statistical significance between a given parameter in test plants and controls. NS, not significant; ^*^indicates values of *p* < 0.0001.

**Figure 4:**
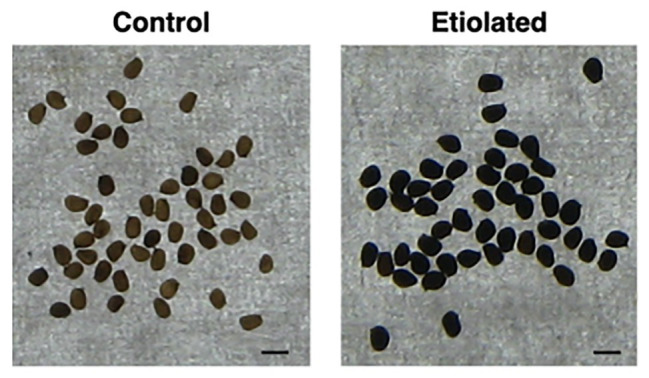
Dimension and morphology of seeds from controls and etiolated capsules. Seeds from etiolated capsules were found to be evidently bigger when compared to controls. Moreover, differences in pigmentation sometimes appeared as an evident feature. The scale bar at the bottom-right indicates 1 mm.

Except for the etiolation, the two types of capsules did not show any difference in development and shape (Figure 2). Accordingly, we investigated whether the etiolation could affect the fitness and the quality of seeds. For both samples, dried capsules, seeds, and empty capsules (obtained after careful removal of the whole seeds) were weighed finding that the full dark-developed capsules were ~24% heavier (Supplementary Table 1; Figure 3). This difference was mainly attributed to the seeds since empty-etiolated capsules were only exceeding the weight of the controls by ~2.05% (Supplementary Table 1; Figure 3), while the seeds total mass was ~50% higher in seeds from etiolated capsules (Supplementary Table 1; Figure 3). Next, we attempted to investigate whether the difference in seeds’ mass was due either to their number, density, or both. Dimensionally, seeds from etiolated capsules were ~26.70 and ~33.16% bigger in length and width, respectively, and the two types of seeds had an almost constant ratio of ~1.3 for each dimension (length 996/786 = 1.27; width 720/541 = 1.33; Supplementary Table 1). Interestingly, these values suggested that, if the seed shape is approximated to the one of an ellipsoid, and the 1.3 ratio is assumed to be valid also for the third dimension (thickness), then the volume of seeds from etiolated capsules can be estimated as ~2.2 times bigger than the one of controls. On the other hand, the weight of the seeds was found to be increased ~41% (~0.41 times) with respect to controls (Supplementary Table 1; Figure 3). Accordingly, dimensions and morphology between the two types of seeds differed significantly finding them increased with respect to the volume and decreased with respect to the density. Finally, a difference in pigmentation between the two types of seeds sometimes appeared as an evident feature, but not always obvious (Figure 4).

### Seeds of Etiolated Capsules Have a Reduced Dormancy

**Figure 5:**
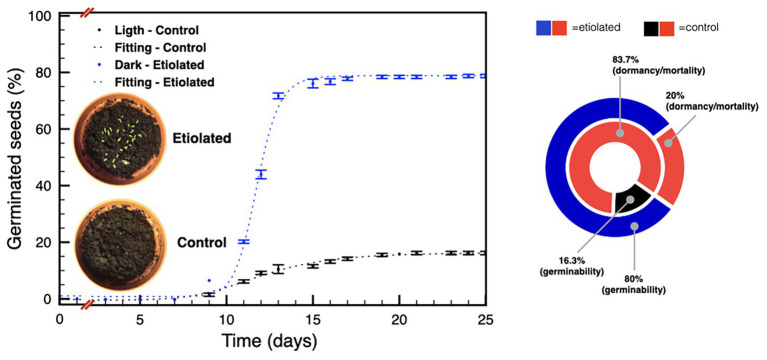
Germination tests on seeds 30 DPA. The plot shows the germination speed of both groups of seeds; the diagram summarizes the plot results and indicates the dormancy/mortality extent in both groups. Data were expressed as mean ± SD of three replicates.

Given the considerable differences in weight between seeds from etiolated and control capsules, we investigated any physiological difference between the two pools. We started this part of the study considering the known decay of seeds dormancy within time in *N. tabacum* L. (Leubner-Metzger, 2005; Finch-Savage and Leubner-Metzger, 2006), and the declined germinability after 6 months of storage without any priming treatment (Min, 2001). Accordingly, germination tests were performed on 30 and 90 DPA seeds. Experiments performed on 30 DPA seeds evidenced a typical primary dormancy of the controls (Figure 5) with a percentage of germinability of ~16% (Figure 5), while, unexpectedly, seeds from etiolated capsules did not show any reluctance to germinate with final rates of ~80% (Figure 5).

**Figure 6:**
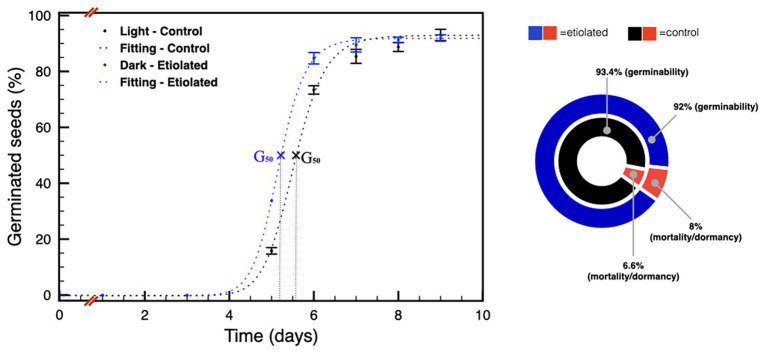
Germination tests on seeds 90 DPA. The plot shows the germination speed of both groups of seeds; the diagram summarizes the plot results and indicates the dormancy/mortality extent in both groups. Data were expressed as mean ± SD of three replicates.

These differences were much less pronounced on 90 DPA seeds (Figure 6), and the difference in the level of dormancy appeared slightly inverted respect to the previous comparison between the 30 DPA seeds. Considering the germination capabilities of the 30 DPA seeds from etiolated capsules, the same seeds type at 90 DPA showed a further reduced physiological dormancy, in agreement with previous evidence (Leubner-Metzger, 2005; Finch-Savage and Leubner-Metzger, 2006). This observation was also evidenced by their prompter ability to emit the embryonic root with respect to controls (data not shown). In particular, for 90 DPA seeds, the time needed for 50% of seeds to germinate (G_50_) is reached by seeds from etiolated capsules at less than half a day before the one from controls (Figure 6). Finally, 90 DPA seeds from etiolated capsules were also found to be less vital, carrying a reluctance to germinate, due to mortality and/or dormancy, equal to ~8%, which is a higher value when compared to the ~7% of the 90 DPA seeds from controls (Supplementary Table 1; Figure 6).

### Seeds From Etiolated Capsules Carry Altered Levels of Hormones and Their Metabolites

Finally, we analyzed the concentration of the phytohormones and their metabolites in 45 DPA seeds collected from both etiolated and control capsules. Except for gibberellins (GA_1_), which were found to be less concentrated with respect to controls, the whole phytohormones were increased in seeds from etiolated capsules (Table 2). In particular, auxins (*IAA* and *IAA*-*Asp*) and cytokinins (cZ and *cZR*) were found to be present with concentrations 2 and 3.5 times higher, respectively, when compared to controls. This tendency was also observed for the inhibitory hormone abscisic acid (ABA), even if less pronounced (1.6 times). As already mentioned, the level of gibberellins was found to be exceptionally low in seeds from etiolated capsules, especially when considering the already low levels normally found in controls (seeds under dormancy).

**Table 2:** Phytohormones levels for 45 day post-anthesis (DPA) seed from etiolated capsules (dark) and control capsules (light).

Phytohormone	Light (ng/g)	Dark (ng/g)	Δ% respect to controls
SA^**^	54.937 ± 2.021	83.681 ± 6.853	+52.31
IAA-Asp^***^	28.066 ± 4.789	97.313 ± 1.553	+246.67
ABA^***^	26.062 ± 1.259	41.752 ± 1.878	+60.21
IAA^**^	12.336 ± 1.437	29.526 ± 2.890	+139.3
GA_1_^╪^	6.542 ± 0.192	<0.8	-
OPDA^**^	4.803 ± 0.393	9.437 ± 0.809	+96.56
JA-ILE^**^	1.069 ± 0.025	1.514 ± 0.091	+40.65
JA^***^	1.037 ± 0.048	1.484 ± 0.021	+41.83
cZR^***^	0.201 ± 0.009	0.521 ± 0.049	+157.5
cZ^*^	0.013 ± 0.005	0.028 ± 0.004	+150

Values are mean ± SD of three replicates. The *p* indicate statistical significance between the levels of a given hormone in seeds from etiolated capsules with respect to controls.

* Indicates values of *p* < 0.05, for each phytohormone.

** Indicates values of *p* < 0.005, for each phytohormone.

*** Indicates values of *p* < 0.0005, for each phytohormone.

╪ The LOD of GA_1_ is 0.8 nM.

Among other differences, seeds from etiolated capsules carried levels of auxins (IAA-Asp) much higher (more than double amounts) than ABA. On the contrary, these two phytohormones (IAA-Asp and ABA) had almost equivalent values in seeds from control capsules. Taking into account the reduced levels of gibberellins, these differences explained the absence of dormancy in seeds from etiolated capsules, and hence, suggested that the auxins levels might be primarily involved in the anomalous properties of seeds.

Similarly, also other phytohormones and their metabolites such as OPDA, JA, JA-ILE, and SA were found with higher levels on seeds from etiolated capsules when compared to controls (Table 2).

## Discussion

The autotrophic metabolism characterizes the whole first stage of fruit differentiation and ripening, and persists until the advanced stages of veraison (White, 2002; Carrari and Fernie, 2006; Prasanna et al., 2007). In most of the fruits, the end of this long process becomes evident by the change from green to the typical colors of a given species (White, 2002; Prasanna et al., 2007). As a metabolic implication of these events, fruits turn their metabolism from autotrophic to heterotrophic indicating that the phenological stage of veraison is reached (Perkins-Veazie, 1995; Trainotti et al., 1999; Aharoni and O’Connell, 2002; Blanco-Portales et al., 2002; Benìtez-Burraco et al., 2003; Collu et al., 2017). From this moment, a final hormone-mediated stage of senescence takes place, and fruits are subjected to an intense recycle of substances by mobilizing them to the rest of the plant while accumulating antimicrobial components and pigments to which a primary function of antioxidants is associated (Sun et al., 2018; Piano et al., 2019).

The advanced stages of ripening have been deeply studied, and their control mechanisms are mediated by phytohormones (Kumar et al., 2014; Robert, 2019). In particular, fruit ripening is regulated by ethylene, while seeds dormancy, which is essential for the embryonic differentiation, is regulated by a fine balance between ABA and gibberellins, or/and auxins (Kucera et al., 2005; Weiss and Ori, 2007; Sreenivasulu, 2017; Figueiredo and Köhler, 2018). Contrary to the ripening and its heterotrophic aspects, the first stages of fruit development and its autotrophic phase are less described. This is particularly true with respect to the metabolic contribution of fruit photosynthesis to both, seed development and fruit growth. To understand the extent of the own photosynthetic contribution in fruits, we have studied the growth of *N. tabacum* capsules in absence of light by inducing etiolation in fruits. Under these growth conditions, we have looked at the main observable differences in photosynthetic parameters, the mass of carpels, the mass of seeds, dimensions of seeds, germinability and mortality of seeds, and phytohormones levels. Dark-developed tobacco capsules underwent to etiolation with the typical exception of the receptacle region, which maintained the characteristic green color indicating the persistency of the photosynthetic parenchyma (Figures 1, 2). This aspect suggests the presence of different regulation mechanisms standing behind these two anatomical parts of the fruit when the fruit itself is subjected to a prolonged dark period. In association with the reduction of the photosynthetic parenchyma, it was observed a significant increase in the weight of seeds from etiolated capsules (Supplementary Table 1; Figure 3). These striking differences can be interpreted as evidence for increased sink properties of the etiolated fruits in a condition, where fruits are not able to perform their own photosynthesis (Supplementary Table 1; Figure 3). Hence, under normal light conditions, photosynthates originate not only from elsewhere sources in the plant (leaves) but also from the fruit itself. These observations are consistent with the Münch scheme according to which the mass of photosynthates (*P*) moves through the phloem as a result of the difference in solute concentration between the sink, fruits, and the source, leaves (Christy and Ferrier, 1973). In the case of etiolated capsules, the absence of photosynthates production causes an increased difference in concentration with respect to the sources. This is evidently correlated with the difference in seeds mass; accordingly, we propose that the ratio between seeds mass from controls and etiolated capsules (m_c_/m_e_) can be reasonably assumed as an approximation of the ratio between the differences in photosynthates concentrations for the same conditions (Δ[*P*]_c_/Δ[*P*]_e_). This assumption can be described by the following formula Δ[*P*]_c_/Δ[*P*]_e_ = m_c_/m_e_. In this respect, it is possible to estimate the contribution of the capsule in photosynthates production under normal light conditions. In fact, the Δ[*P*]_c_ can be expressed as the difference between the solute concentration in the source (C), which is strictly depending on the photosynthetic rate on leaves and assumed to be constant in both conditions, and the solute concentration in the sink, which can be considered composed of two components: a basal one (B) and a photosynthetic one (A), so that Δ[*P*]_c_ = C − B − A. Similarly, for the etiolated conditions, it is valid that Δ[*P*]_e_ = C − B, where A, in this case, is missing due to the absence of the photosynthetic activity in etiolated capsules. Accordingly, the equation becomes Δ[*P*]_c_/Δ[*P*]_e_ = m_c_/m_e_ = C − B − A/C − B. From this equation can be obtained the photosynthetic contribution as A = (C − B) [1 − (m_c_/m_e_)] = Δ[*P*]_e_ [1 − (m_c_/m_e_)]. This equation gives an indicative value of the fruit contribution in presence of light, and it uses the etiolated condition (Δ[*P*]_e_) as a reference. Certainly, this value is characteristic of specific experimental conditions (e.g., light conditions) and species studied, thus it should be found empirically for each case. In our study, if we consider the seeds mass shown in Supplementary Table 1, the component [1 − (m_c_/m_e_)] assumes a value of [1 − (61.5/92.5)] = 0.3351. It means that, here, due to the photosynthetic contribution of the capsule (A), the value Δ[*P*]_c_ will decrease of 33% in etiolated capsules. It is worth to stress that we assume a constant basal contribution (B) that is not affected by light itself, but it is only related with the basal metabolism and is equivalent to the metabolic rate in absence of light (where only respiration takes place and CO_2_ is emitted). This is an important detail which Reich et al. have elegantly shown to be only depending on the nitrogen availability (Reich et al., 2006).

Seeds from dark-developed capsules have not only a difference in mass but also in their tendency to germinate; in fact, they show an almost absent dormancy, as observable by their peculiarity to germinate immediately after the senescence of the fruit (Figure 5). The effect of environmental factors, in particular temperature, light, and age, on seed germinability/dormancy is well documented (Bhatt et al., 2016). These effects are also known to be reflected endogenously as changes in phytohormones levels, especially ABA and GA, that finally result in different timing of seed germinability/dormancy (Chen et al., 2020). These properties are in line with what is observed in the present work, where light affects seeds maturity/maturation with a hormone-mediated mechanism.

The characteristically reduced dormancy of seeds from etiolated capsules was associated with a counterintuitive presence of GA_1_ levels much lower when compared to ABA levels, which, on the contrary, are significantly increased when compared to controls (Table 2). These differences suggest that a common but opposite regulation system acts on genes related to the biosynthesis of both these phytohormones affecting their levels. Along the same line, “dark-developed” seeds carried also a significantly increased level of auxins when compared to controls (Table 2). The ratio between total auxins level, with particular reference to the most common IAA and *IAA*-*Asp*, and the ABA, is ~3 times higher in seeds from etiolated capsules with respect to a value of ~1.5 times in controls. This striking difference allows us to attribute to auxins the anomalous germination properties of dark-developed seeds. In this respect, it is important to mention that *IAA*, the free and active form, is in equilibrium with IAA-Asp, which is, especially in the seed, functioning as a reservoir that can be promptly released as soon as the levels of free IAA decrease. This steady-state equilibrium, based on the conjugated form as a reservoir (Ljung et al., 2002; Seidel et al., 2006), would guaranty a constant conversion to IAA keeping efficiently high its levels. Such a scheme would be consistent with the typical IAA properties of attracting nutrients (Ferguson and Beveridge, 2009), a fact that would also explain the results shown in Figure 3 and Supplementary Table 1.

Similarly, also the main cytokinins, in particular *cZ* and *cZR*, increased their levels in dark-developed seeds. This effect could be considered in the frame of the increased sink properties of dark-developed seeds to which the phloem would bring not only nutrients but also cytokinins (Jameson and Song, 2016). Accordingly, being the level of phytohormones the main difference between the two types of seeds, these differences are expected to influence the embryos differently explaining the observed germination profiles (Figures 5, 6).

Fruits are centers of photosynthates accumulation, and this sink-organ property is essential for allowing their optimal development. However, young fruits have photosynthetic parenchyma that let them perform photosynthesis; thus, they behave as source organs, allowing proper development of the seed (Lytovchenko et al., 2011). In the present study, the dual role of tobacco fruits as sink-and-source organs evidently emerged by showing how the flower, first, and the fruit, after, behaves as a hormone-mediated self-regulating center able to balance its metabolic rate by a variable supply of external photosynthates sources. In fact, the induced etiolation of fruits is associated with the increased mass of seeds and ovaries, suggesting the presence of two sources of photosynthates: (i) the fruit itself, which is not working under etiolation, and (ii) the leaves, which compensate the absence of photosynthesis in etiolated fruits. Under etiolation, fruits cannot behave as a source organs, and their missing contribution is compensated by the other centers, mainly leaves. In line with the Münch theory (Christy and Ferrier, 1973), this implies a delayed retro-regulation due to the long distance between sources and sink and a stronger inertial effect driven by the bigger difference of solutes concentration between leaves and flowers. On the contrary, under normal light conditions, the fruit behaves as source organ and drives the photosynthates accumulation, getting a fast response and contributing significantly to the production and storage (Ruan et al., 1997; Patrick and Offler, 2001; Lalonde et al., 2003). Interestingly, considering the storage of photosynthates with respect to the observed decreased density of seeds, this feature can be explained with the tendency to store starch and glucans with different branching properties, as frequently observed under stress conditions (MacNeill et al., 2017).

Moreover, the contribution of sepals, shown to not etiolate even when the whole flower is covered, should be also taken into account. The absence of etiolation in sepals suggests a regulation mechanism rather linked with leaves than with the capsule, ultimately implying that the mechanism described above for leaves is most likely valid also for this part of the flower. Furthermore, in tobacco plants, sepals are reported to remain green in all the flower developmental stages, to have the level of chlorophyll similar to those of leaves, and timing of senescence different than in petals (Müller et al., 2010). This last observation might also be valid for the etiolation mechanism, ultimately keeping green the sepals even in the absence of light. However, the fine contribution of sepals, which is difficult to study due to their reduced dimensions, remains to be elucidated, especially in terms of photosynthetic rates.

The dynamics and regulation mechanisms related to the development of fruits and seeds are essential not only for understanding the extent of the sink/source behavior in fruits with respect to other source organs but also for future applications aimed at implementing seeds quality and stability as well as fruits production. The present study evidenced the relevancy of the heterotrophic phase of fruits in contributing to reach the final stage of their ripening and maturity.

## Data Availability Statement

All datasets presented in this study are included in the article/Supplementary Material.

## Author Contributions

DP conceived the study, participated in its design and coordination, performed the experimental measurements, processed the experimental data, interpreted the data, and drafted and revised the manuscript. DF participated in the design and coordination of the study, performed the experimental measurements, interpreted the data, and drafted and revised the manuscript. PH contributed to interpreting the data and helped in drafting the manuscript. AA and PC contributed to interpreting the results, and helped in drafting the manuscript. EC and MK helped in analyzing seeds morphology and dimensions and in revising the manuscript. ML participated in the design and coordination of the study and helped in drafting the manuscript. All authors contributed to the article and approved the submitted version.

### Conflict of Interest

The authors declare that the research was conducted in the absence of any commercial or financial relationships that could be construed as a potential conflict of interest.

## Footnotes

^1^https://mycurvefit.com/

^2^https://apps.micw.org/apps/plot2/index.php

^3^https://www.graphpad.com/quickcalcs/ttest1.cfm

## Abbreviations

ABA: Abscisic acid
cZ: Cis-Zeatin
cZR: Cis-Zeatin-9-riboside
D2-GA_1_: Giberellin-d2
D2-JA: Jasmonic-2,4,4-d3-(acetyl-2,2-d2) acid
D4-SA: Salicylate-d4
D5-IAA: Indole-2,4,5,6,7-d5-3-acetic acid
D6-ABA: Abscisic acid-d6
DPA: Day post-anthesis
GA_1_: Gibberellins
IAA: Indole-3-acetic acid
IAA-Asp: Indole-3-acetylaspartic acid
JA: Jasmonate
JA-ILE: Jasmonoyl isoleucine
OPDA: 12-oxo-phytodienoic acid
PSII: Photosystem II
SA: Salicylate
