# Supplementary material for: The Impact of Fruit Etiolation on Quality of Seeds in Tobacco
Source: Front Plant Sci. 2020 Oct 8;11:563971. doi: 10.3389/fpls.2020.563971 (PMC7578389; doi:10.3389/fpls.2020.563971)
Supplement: Supplementary file 1 [file Data_Sheet_1.pdf]

Supplementary Table 1

	Dark	Light (control)	Dark vs Light
**Full capsules mass (mg)***	165.7 ± 8.8	134.1 ± 5.1	23.56%
**Seed mass per capsule (mg)***	92.5 ± 6.0	61.5 ± 5.3	50.41%
**Seed mass average on 50 seeds (µg)***	49 ± 3.8	34.7 ± 2.1	41.21%
**Empty capsules mass (mg)^{NS}**	69.7 ± 4.5	68.3 ± 3.2	2.05%
**Seed length (µm)***	996 ± 48	786 ± 51	26.70%
**Seed width (µm)***	720 ± 51	541 ± 46	33.16%
**Germinability 30 DPA (%)***	80 ± 1	16.3 ± 0.58	500
**Germinability 90 DPA (%)***	92 ± 1	93.4 ± 1.5	-1.39

Sup. Table 1. Measurements of etiolated capsules, their seeds and comparison with controls. Data were expressed as mean ± SD on 9 capsules from 3 different plants. P values indicate statistical significance for a given measurement between etiolated and control capsules. NS, Not Significant; Symbol * indicates a p-value < 0.0001 between etiolated and control capsules for a given parameter.
